# Diagnostic utility of an automated fundus-OCT camera in the emergency department: a retrospective review

**DOI:** 10.1186/s12886-025-04228-z

**Published:** 2025-07-18

**Authors:** Samyuktha Guttha, Andrew M. Huang, Kiran Malhotra, Andrea L. Blitzer

**Affiliations:** 1 NYU Department of Ophthalmology, 222 E 41 st St, 4F, New York, NY 10016 USA; 2 NYU Department of Clinical Informatics, 550 1 st Ave, New York, NY 10016 USA

**Keywords:** Automated fundus-OCT camera, Emergency department, Point-of-care retinal imaging

## Abstract

**Background:**

Posterior segment pathology can be challenging to diagnose and may lead to irreversible vision loss. Ocular imaging modalities are limited in the emergency department (ED) where many posterior segment emergencies present. Automated fundus-OCT cameras are emerging as a rapid, user-friendly and cost-effective tool in the ED.

**Methods:**

Fundus and OCT images were taken by residents as needed for patients undergoing ophthalmology consultation in an academic ED in 2023. Image type and quality were graded on a scale of 1 to 3 (poor, adequate, good). Medical records were reviewed to record relevant patient characteristics. Statistical analysis was performed using unequal variances T-test to compare patients with poor and at least adequate photo quality.

**Results:**

288 patients were imaged with the fundus-OCT camera over 12 months. Camera utilization increased at the start of the academic year, then decreased towards the end of the academic year. Adequate diagnostic quality images were taken in 92% of color photos and 94% of OCT images. The odds of poor image quality were significantly higher in patients presenting with logMAR > 1.0 (OR 8.07, 95% CI 3.28–19.86, *p* < 0.001) and age > 65 (OR 4.69, 95% CI 1.94–11.34, *p* < 0.001).

**Conclusions:**

Fundus photography and OCT are emerging as viable imaging modalities in the ED where access to ophthalmic expertise and equipment has traditionally been limited. Automated fundus-OCT cameras can offer high quality images that may facilitate rapid and accurate diagnosis of posterior segment pathology.

## Background

Ocular emergencies account for up to 2% of all patient encounters in the emergency department (ED) or approximately 2 million visits annually in the United States [1, 2]. Posterior segment pathology can be challenging for emergency physicians to diagnose and may lead to irreversible vision loss if not managed in a timely fashion. When suspected, patients should undergo comprehensive ophthalmic evaluation including slit lamp biomicroscopy, fundoscopy, and appropriate imaging studies.

However, few ocular imaging modalities are readily available in the emergency setting. While CT and MRI machines are ubiquitous, their diagnostic yield is mostly limited to neuro-ophthalmic and orbital disease [3]. Despite their limited utility in the diagnosis of ophthalmic conditions, CT and MRI studies in eye-related ED visits have nearly doubled in the span of a decade, far outpacing the growth in visit volume [4]. They are often ordered unnecessarily and contribute to excessive utilization of healthcare resources. Alternatively, point-of-care ocular ultrasound can be used to diagnose certain posterior pathology with high sensitivity and specificity, but is highly dependent on user ability [5, 6]. As the use and diagnostic yield of imaging has increased in the ophthalmology clinical setting, there is a lack of ocular imaging modalities in the ED that are rapid, user-friendly and cost-effective.

Recently, fundus photography and optical coherence tomography (OCT) have shown promise when introduced into the emergency setting [7–10]. These retinal imaging modalities are considered standard of care in ophthalmology clinics, but have traditionally been limited to the outpatient setting due to cost, portability, and the need for experienced technicians. With modern advances in technology, combined fundus-OCT cameras have been developed that are fully automated and relatively low cost. These cameras are beginning to be placed in EDs given their potential use in screening exams, teleophthalmology consults, and stroke evaluations [11–13]. Recently, we introduced an automated fundus-OCT camera into a busy tertiary-care academic ED to provide ancillary ocular imaging for ophthalmology consults. This study reviews the first year of our experience and assesses the diagnostic utility of point-of-care retinal imaging in the ED.

## Methods

This study is a retrospective chart review of all patients who were evaluated by the ophthalmology service and imaged with the fundus-OCT camera between January 1, 2023 and December 31, 2023 at the NYU Langone Health Manhattan ED. Research was conducted in accordance with the tenets of the Declaration of Helsinki and HIPAA regulations. The review was undertaken as a quality improvement initiative and does not constitute human subjects research.

Ophthalmology residents underwent a training session to learn how to operate the automated fundus-OCT camera (Topcon Maestro2, Topcon Corporation, Tokyo, Japan). The camera is capable of taking fundus photos of the posterior pole and retinal periphery as well as OCT of the anterior segment, macula, and retinal nerve fiber layer. OCT and fundus photos of the posterior pole could be simultaneously acquired and registered. The camera uses robotic technology to automatically align, focus, and capture images with a single touch, requiring minimal operator input and patient instruction. The camera’s small form factor allows it to be placed in the corner of the ED eye room. Images were automatically saved to the manufacturer’s proprietary image filing system (IMAGEnet6).

Patients presenting to the ED with urgent eye complaints requiring ophthalmology consultation were evaluated by the ophthalmology on-call resident which included both junior and senior residents. The resident completed their evaluation with the option of using the fundus-OCT camera at their own discretion. There was no requirement to use the camera except in cases of suspected central retinal artery occlusion (CRAO).

The IMAGEnet6 camera database was used to identify patients imaged between January 1, 2023 and December 31, 2023. The associated patient charts and ophthalmology consult notes were reviewed to identify patient demographics, date of encounter, presenting near visual acuity, chief complaint, and diagnosis. Cases were excluded if images were not able to be linked to a patient chart. For each case, the type of imaging performed was recorded, and subjective image quality was graded by a single author on a scale of 1 to 3. A score of 1 indicated poor quality image with no useful diagnostic features, a score of 2 indicated adequate image quality with identifiable structures and lesions, and a score of 3 indicated good quality images with clearly visible fundus details. If duplicate images of the same type were present, the highest quality image was chosen to represent the set. Descriptive statistics was used to summarize patient characteristics. Variables associated with poor image quality were assessed by calculating odds ratios and corresponding 95% confidence intervals using contingency table analysis and Fisher exact test.

## Results

### Patient characteristics

From January to December 2023, 316 patients were imaged with the camera. During the study period a total of 646 ophthalmology consults were identified via orders placed by the ED, however, this does not include a large number of consults carried out without a linked order. 28 patients were excluded due to insufficient identifying information to link to a medical record or ophthalmology consult order. Table 1 shows the demographics of the total 288 patients included in the study. 127 (44.1%) were men and 161 (55.9%) were women with a mean age of 49.9 ± 21.0 (range 4–93). Represented races include White (53.5%), Black (20.5%), Hispanic (8.7%), Asian (12.1%), and other backgrounds (5.2%). The mean logMAR near visual acuity on presentation was 0.4 ± 0.7 (Snellen equivalent 20/50).

**Table 1:** Patient characteristics

Total patients	288
Age, mean ± SD	49.9 ± 21.0
Sex, n (%)	
Male	127 (44.1)
Female	161 (55.9)
Race, n (%)	
White	154 (53.5)
Black	59 (20.5)
Asian	35 (12.2)
Hispanic	25 (8.7)
Other/unidentified	15 (5.2)
Presenting logMAR, mean ± SD	0.4 ± 0.7
0.0–0.3 (20/20–20/40)	207 (72.4)
0.4–0.9 (20/50 − 20/160)	22 (7.7)
1.0–1.3 (20/200 − 20/400)	28 (9.8)
1.4–3.0 (20/400 - NLP)	29 (10.1)
Unable due to mental status	2 (0.7)

### Image type and quality

**Fig. 1:**
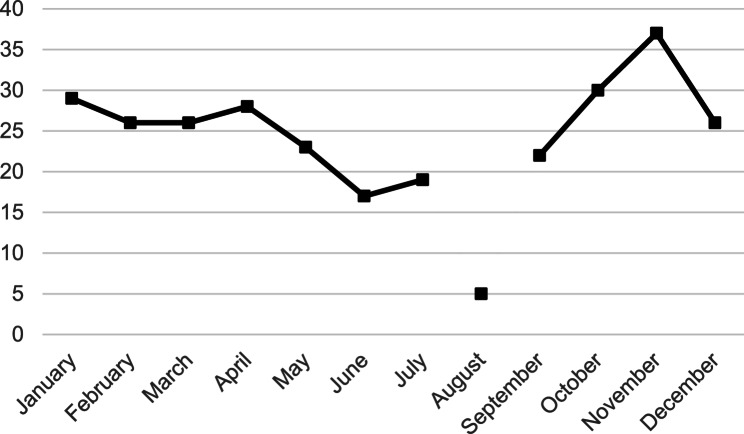
Number of patients imaged per month. *August was an outlier due to 3 weeks of camera malfunction

**Fig. 2:**
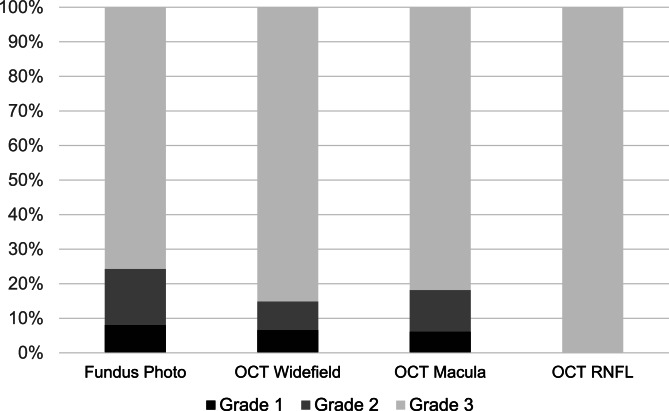
Image Quality

A mean of 24 patients were imaged with the camera per month (range 5–37). There was an upward trend in camera utilization at the start of the academic year from July to November, and a downward trend from December to June (Fig. 1). Only 5 images were taken in August due to camera malfunction for approximately 3 weeks. Both color fundus photos (*n* = 284, 98.6%) and OCT (*n* = 278, 96.5%) were taken for nearly all patients. The 4 patients who did not have a fundus photo taken were instead imaged with anterior segment OCT (*n* = 4, 1.4%) to evaluate the iridocorneal angle and corneal pathology. Mean image quality was 2.68 ± 0.62 for fundus photos (*n* = 284), 2.79 ± 0.55 for OCT widefield (*n* = 229), 2.76 ± 0.56 for OCT macula (*n* = 33), and 3.00 ± 0 for OCT RNFL (*n* = 12). Figure 2 shows that 91.9% of color photos and 93.8% of OCT images were of at least adequate quality (grade 2 or 3). The odds of poor image quality were significantly higher in patients presenting with visual acuity logMAR > 1.0 (OR 8.07, 95% CI 3.28–19.86, *p* < 0.001) and age > 65 (OR 4.69, 95% CI 1.94–11.34, *p* < 0.001) (Table 2). Presentation to the ED in the early months of the academic year (July to September) was not associated with poor image quality (OR 1.13, 95% CI 0.37–3.49, *p* = 0.83).

**Table 2:** Association between presenting variables and poor image quality

Variable	Odds ratio	95% CI	*P* value
Age ≥ 65	4.69	1.94–11.34	< 0.001
VA logMAR ≥ 1.0 (Snellen 20/200)	8.07	3.28–19.86	< 0.001
Early in residency (July – September)	1.13	0.37–3.49	0.83

### Most common diagnoses

Table 3 summarizes the most common diagnoses imaged with the camera. Imaging was most frequently used to evaluate pathology of the vitreoretinal interface (50, 17.4%) and optic nerve (44, 15.3%). Imaging helped rule out posterior pathology in 93 patients (32%) who presented with eye-related complaints with no acute posterior segment findings on exam (i.e. transient vision loss or ocular surface disease).

**Table 3:** Most common diagnoses imaged

Diagnosis	*N*	%
No posterior pathology	93	32.3%
Anterior pathology	65	22.6%
Transient vision loss	28	9.7%
Vitreoretinal interface disorders	50	17.4%
Posterior vitreous detachment	33	11.5%
Retinal tear	5	1.7%
Fovea-on retinal detachment	7	2.4%
Fovea-off retinal detachment	5	1.7%
Optic nerve pathology	44	15.3%
Papilledema or idiopathic intracranial hypertension	19	6.6%
Optic neuritis	10	3.5%
Other optic neuropathy (includes NAION)	6	2.1%
Open angle glaucoma	6	2.1%
Angle closure glaucoma	3	1.0%
Macular pathology	26	9.0%
Proliferative retinopathy	9	3.1%
Nonproliferative retinopathy	5	1.7%
Macular edema or subretinal fluid	5	1.7%
Other maculopathy	7	2.4%
Vascular Occlusion	18	6.3%
Central retinal artery occlusion	5	1.7%
Branch retinal artery occlusion	5	1.7%
Central retinal vein occlusion	8	2.8%
Uveitis	19	6.6%
Anterior uveitis	7	2.4%
Posterior or panuveitis	10	3.5%
Infectious uveitis	2	0.7%
Neuro-ophthalmic disorders	20	6.9%
Traumatic disorders	13	4.5%
Orbital and adnexal disorders	5	1.7%

## Discussion

Fundus photography and OCT are emerging as viable imaging modalities in the ED where access to ophthalmic expertise and equipment has traditionally been limited. In this study, an automated fundus-OCT camera was utilized as needed by ophthalmology residents for ED consults. We report the most common indications for which the camera was used and show its capability to acquire high-quality fundus and OCT images in an academic ED.

The majority of patients imaged had no posterior segment pathology (32.3%), which helped to rule out retinal or optic nerve disorders. In many academic institutions, junior residents are the front line of the ophthalmology consult service with direct supervision provided only for a minority (1–30%) of ED consults [14]. Understandably, junior residents who are beginning to take primary call may not yet be confident in their fundoscopy skills and interpretation. They may be more reliant on retinal imaging to aid or confirm diagnoses, rule out pathology, document exam findings at time of presentation, and communicate findings with patients and supervising physicians. In this study, residents imaged patients at their own discretion, which suggests the camera provided an incremental benefit in certain encounters. For example, in 28 cases of transient vision loss, a normal fundus photo and OCT helped confirm that subtle findings such as retinal emboli were not overlooked on exam. The frequency of imaging tended to increase over the first few months of the academic year from July to November and then decreased towards the end of the year. We hypothesize that imaging frequency increased as first year residents became familiar with the camera, then decreased as they became more confident and independent in their clinical exams.

In the United States, the most common posterior segment pathologies that present to the ED are posterior vitreous detachment and retinal detachment [2, 15]. Similarly, the most common diagnoses made with the fundus-OCT camera were related to the vitreoretinal interface (17.4%). Widefield OCT scans of the optic disc and macula can be used to determine PVD status [16–18] with some studies showing higher rates of PVD identification on OCT compared to biomicroscopy [19]. – [20] In patients with acute flashes and floaters, hyperreflective vitreous opacities on OCT akin to Shafer’s sign have 65–100% sensitivity and 65–94% specificity in diagnosing retinal tears [21–23]. The lack of this finding thus has a strong negative predictive value for a retinal break, especially as an adjunct to clinical exam. For the 12 patients with retinal detachment, imaging was used to determine the extent of foveal involvement and posterior hyaloid position, which are critical considerations for preoperative surgical planning and prognostication. OCT offers axial resolution far greater than that achieved by biomicroscopy to distinguish between fovea-on, fovea-off, and even fovea-splitting detachments. Finally, retinal tears and detachments are a leading cause of misdiagnosis-related malpractice claims in ophthalmology [24]. Imaging not only facilitates diagnosis but provides photo documentation to support decision making. This may be of particular interest to academic centers with trainees taking primary call.

**Fig. 3:**
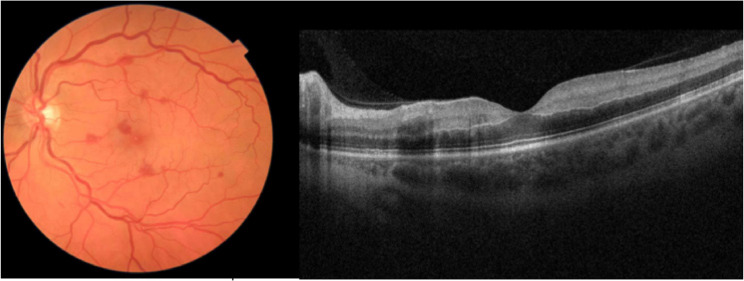
CRVO with paracentral acute middle maculopathy masquerading as CRAO. Fundus photo and OCT in a patient diagnosed with central retinal vein occlusion with paracentralacute middle maculopathy. Clinical presentation and hyperreflectivity of inner retinal layers onOCT may mimic a CRAO

Recently, a novel stroke protocol based on remote OCT interpretation has been proposed to improve outcomes in patients presenting with central retinal artery occlusion (CRAO) [13]. These patients are eligible for thrombolytic therapy within 4.5 to 6 h of symptom onset but often have delayed presentation. Even among patients who present promptly, only a minority (1/3) receive ophthalmic evaluation in time before the therapeutic window closes [25]. Automated fundus-OCT imaging offers several advantages that can reduce the diagnostic time gap from hours to minutes: (1) non-mydriatic cameras do not require time for pupil dilation, (2) images can be reviewed remotely by retina specialists, and (3) OCT has higher accuracy (up to 100% sensitivity and 94% specificity) than clinical exam within the initial hours of CRAO onset [26, 27]. Of the 5 CRAO cases in this study, only 1 presented within 4.5 h and received intra-arterial tissue plasminogen activator (TPA) with visual recovery from hand motion to 20/300. Additionally, we propose that fundus photography is a necessary addition to OCT-only stroke protocols to ensure accurate diagnosis. One patient presented with 6 h of painless vision loss to 20/200 with afferent pupillary defect and inner retinal hyperreflectivity and thickening on OCT. However, the fundus appearance was more consistent with central retinal vein occlusion with paracentral acute middle maculopathy, thus obviating the need for TPA. (Fig. 3). Vision eventually recovered to 20/25 without intervention. The latest camera models now offer OCT-angiography, which may help confirm retinal nonperfusion in equivocal cases [28]. 

Overall, over 90% of fundus photos and OCT images acquired in this study were of good diagnostic quality (grade 2 or 3) comparable to prior studies [7, 29]. Even residents very early in their training were able to obtain good quality images and there was no association with patient presentation in the early months of the academic year (July to September) with poor image quality. Patients presenting with worse visual acuity and older age had an increased odds of poor image quality, possibly due to media opacity or difficulty fixating for the camera. However, we acknowledge that the high image quality achieved in this study may not be generalizable due to selection bias. Since residents examined and dilated most patients prior to imaging, they were able to judge if media opacity or other factors would preclude effective camera use. Photos were often repeated for patients if the initial attempt revealed image artifacts or insufficient quality. Still, we are optimistic about the camera’s ability to acquire high quality images even when operated in less ideal settings by non-ophthalmology providers.

Other limitations of this study include its observational and retrospective design. Ophthalmology residents were not required to use the fundus-OCT camera for all ED consults as this would be too burdensome. There was no clear way of identifying whether the camera directly changed the diagnosis or management for individual cases. Image quality was also subjectively graded by a single author. Future studies can be designed in a self-controlled manner to determine differences in pre- and post-imaging diagnoses. Additional studies are needed to learn how non-ophthalmology providers may utilize the camera in the ED.

## Conclusion

Here we report the most common diagnoses made by ophthalmology residents with the assistance of an automated fundus-OCT camera in the ED. Diagnostic quality fundus photos and OCT images were acquired for over 90% of patients in this cohort. This data supports the feasibility and diagnostic utility of point-of-care retinal imaging in the ED for in-person and tele-ophthalmology consults.

## Data Availability

The datasets used and/or analyzed during the current study are available from the corresponding author on reasonable request.

## Abbreviations

CRAO: Central Retinal Artery Occlusion
CT: Computed Tomography
ED: Emergency Department
HIPAA: Health Insurance Portability and Accountability Act
MRI: Magnetic Resonance Imaging
NLP: No Light Perception
OCT: Optical Coherence Tomography
PVD: Posterior Vitreous Detachment
RNFL: Retinal Nerve Fiber Layer
TPA: Tissue Plasminogen Activator
VA: Visual Acuity
